# Lysyl Oxidase at the Crossroads of Mesenchymal Stem Cells and Epithelial-Mesenchymal Transition

**DOI:** 10.18632/oncotarget.919

**Published:** 2013-03-09

**Authors:** Clémence Thomas, Antoine E. Karnoub

**Affiliations:** Division of Cancer Biology and Angiogenesis, Department of Pathology, Beth Israel Deaconess Medical Center and Harvard Medical School, Boston, MA; Division of Cancer Biology and Angiogenesis, Department of Pathology, Beth Israel Deaconess Medical Center and Harvard Medical School, Boston, MA

Breast carcinoma development is governed by dynamic interactions that operate between mutant cancer cells and the stromal compartments around them, which encompass a collection of mesenchymal cell types, such as endothelial cells, fibroblasts, and immune cells [1]. It is increasingly appreciated that the cancer-associated stroma plays powerful determining roles in breast tumor pathogenesis, particularly in the progression of carcinomas to malignancy. Consequently, a better understanding of the crosstalk operating at the tumor-stroma interface should provide new and viable avenues for anti-neoplastic therapy.

We have previously shown that certain progenitor cells called mesenchymal stem cells (MSCs), which otherwise contribute to the maintenance and regeneration of connective tissues under normal and inflammatory conditions, are also incorporated into the stroma of developing tumors. Most importantly, we showed that the interactions of stromal MSCs with breast cancer cells within primary tumors activate the MSCs, which act back on the cancer cells in a paracrine fashion, increasing their motility, invasion, and metastasis [2]. Although the MSC-based breast cancer metastasis model has been widely adopted, and supported by animal as well as clinical observations [3, 4], we still know relatively little regarding how cancer cells respond to the influences of stromal MSCs.

In an effort to address this question, we analyzed the transcriptome of cancer cells that have been co-cultured with MSCs *in vitro*, and found lysyl oxidase (LOX) to be one of the most prominent features of MSC-activated cancer cells [5]. LOX is a secreted amine oxidase that catalyses collagen and elastin cross-linking in the extracellular matrix, and has been previously shown to regulate breast cancer metastasis [6]. A direct function for LOX in mediating MSC-induced malignancy, however, had not been described.

**Figure 1:**
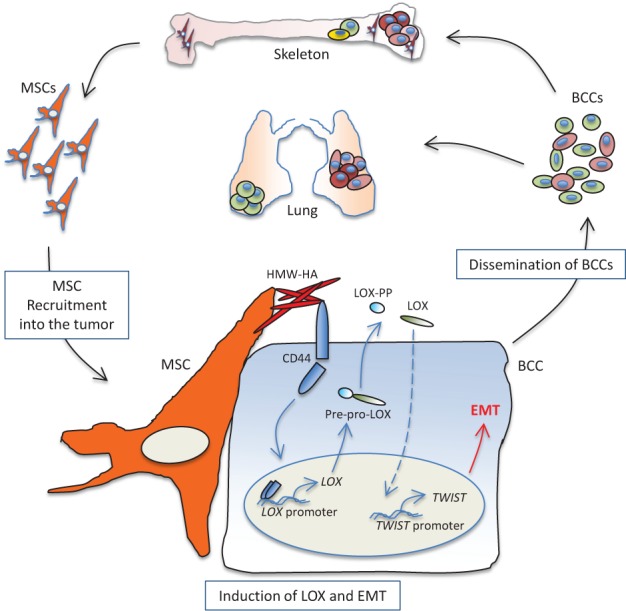
Education of cancer cells by stromal MSCs MSC-derived high molecular weight (HMW) HA causes enrichment of CD44 in the nucleus of the cancer cells, which associates with the LOX promoter, driving its transcription. Immature LOX is secreted to the extracellular space where it is processed into the mature 32kDa form (LOX) and the 19kDa pro-LOX (LOX-PP). Active LOX then drives a cascade of signalling events that lead to Twist induction, onset of EMT, and cancer cell dissemination.

Our investigations showed that LOX over-expression by cancer cells indeed promoted their aggressive phenotypes, such as resistance to anoikis, as well as increased motility and invasion. Importantly, we established that LOX enabled cancer cells to metastasize to bone, suggesting it played important roles in regulating the homing of cancer cells to and/or their colonization of the skeleton. Furthermore, we found that LOX caused robust induction of epithelial-mesenchymal transition (EMT) in the cancer cells, primarily by inducing the expression of the master transcription factor Twist. Of interest is that MSC-induced EMT in the primed cancer cells required LOX expression, which was also necessary for Twist induction in this setting, highlighting a pivotal role for LOX in stroma-induced EMT regulation. Surprisingly, how MSCs triggered LOX expression in the cancer cells resided in the actions of two familiar players: the glycosaminoglycan hyaluronan (HA) and the adhesion receptor CD44. Indeed, MSC-derived HA was sufficient and necessary to induce LOX and Twist in the cancer cells, doing so in a manner that depended upon cancer cell expression of CD44. In these respects, MSC-stimulated cancer cells exhibited nuclear enrichment in an ~50 kDa CD44 product, which paralleled our ability to find an association between CD44 and the *LOX* promoter by chromatin-IP (ChIP) analyses. Altogether, our work revealed a novel CD44-dependent mechanism for *LOX* regulation in cancer metastasis (Fig. 1), and highlighted a critical involvement of this pathway in MSC-induced malignancy [5].

The significant role for LOX in regulating EMT prompted us to investigate whether it played similar roles in controlling cancer stem cell (CSC) phenotypes. Indeed, EMT and CSC phenotypes were demonstrated to be closely related, as cells that have undergone EMT were found to be enriched for CSC phenotypes, and CSCs present within primary or established cancer cells were shown to possess features of EMT [7]. Furthermore, MSC-stimulated cancer cells acquired, in addition to EMT phenotypes, CSC characteristics as well [4, 5], offering a unique system to probe the inter-relationship between the signal transduction events controlling both phenotypes. Strikingly, however, LOX was not capable on its own in triggering CSC propagation, nor was it required for the MSC-induced CSC phenotypes, despite its tight control of Twist. This raises the possibility that the upstream stroma-instigated regulatory pathways governing EMT are separate from those determining CSC phenotypes.

Our work raises a number of translational and conceptual questions that are subjects of active investigations. For example, in light of the concerted expression of CD44, LOX, and Twist in metaplastic breast cancers [5], to what extent is this pathway active in other more commonly diagnosed malignant subtypes of breast cancer? This is of increased clinical pertinence, as anti-LOX-based therapy, already promising in experimental models [6], might also prove useful in the management of metastatic disease in the clinic. Similarly, the findings that HA was sufficient in triggering LOX-Twist activation and that hyaluronidase (HYAL) treatment abrogated the ability of MSCs to stimulate EMT in the cancer cells add another dimension to ongoing HYAL-based anti-neoplastic therapy [8], and suggest that such approaches would prove to be clinically advantageous in combating metastatic disease in the setting of breast cancer.

From the conceptual point of view, our observations provide a framework through which one can probe a number of interesting questions pertaining to tumor-stromal interactions. For instance, now that LOX was demonstrated to be a major conduit for MSC-derived signals that foster EMT in breast cancer cells, what are the nodes used by MSCs to cause the propagation of CSC characteristics, and are such signals exclusive of one another, as recent work suggests [7]? Along the same lines, how LOX activation in turn leads to Twist expression has not been determined, and to what extent this involves the activity of other EMT transcription factors remains to be explored. Finally, as LOX induction in the MSC-activated cancer cells was shown to be transient and ostensibly reversible, it would be most interesting to investigate whether MSCs impart on cancer cells any irreversible phenotypes. If so, such stable characteristics might thus represent examples of long-term memory (or footprint) that disseminated cancer cells retain of their interactions with the tumor stroma within primary tumors – footprints that would likely contribute to the ability of such cells to colonize secondary sites. As the interest in the biology of stromal mesenchymal stem cells and how they contribute to cancer pathogenesis is increasing at a fast pace, answers to these questions will undoubtedly unfold before long.
